# A Multicenter Pilot Study Examining the Role of Circulating Tumor Cells as a Blood-Based Tumor Marker in Patients with Extensive Small-Cell Lung Cancer

**DOI:** 10.3389/fonc.2014.00271

**Published:** 2014-10-14

**Authors:** Chao H. Huang, Jo A. Wick, Gurusingham Sitta Sittampalam, Victor Sanjit Nirmalanandhan, Apar Kishor Ganti, Prakash C. Neupane, Stephen K. Williamson, Andrew K. Godwin, Sarah Schmitt, Nora J. Smart, Sarah Spencer, Peter J. Van Veldhuizen

**Affiliations:** ^1^Division of Hematology & Oncology, University of Kansas Medical Center, Westwood, KS, USA; ^2^Subspecialty Medicine, Kansas City VA Medical Center, Kansas City, MO, USA; ^3^Department of Biostatistics, University of Kansas, Kansas City, KS, USA; ^4^Therapeutics for Rare and Neglected Diseases, National Center for Advancing Translational Sciences, National Institutes of Health, Rockville, MD, USA; ^5^Princess Margaret Cancer Center, University Health Network, Toronto, ON, Canada; ^6^VA Nebraska Western Iowa Health Care System, Division of Oncology-Hematology, Department of Internal Medicine, University of Nebraska Medical Center, Omaha, NE, USA; ^7^Department of Pathology and Laboratory Medicine, University of Kansas Medical Center, Kansas City, KS, USA; ^8^Clinical Molecular Oncology Laboratory, University of Kansas Medical Center, Kansas City, KS, USA

**Keywords:** small-cell lung carcinoma, circulating tumor cells, biomarkers, prognosis, extensive stage

## Abstract

**Background:** Small-cell lung cancer (SCLC), a variant of lung cancer marked by early metastases, accounts for 13% of all lung cancers diagnosed in US. Despite high response rates to treatment, it is an aggressive disease with a median survival of 9–11 months for patients with extensive stage (EX-SCLC). Detection of circulating tumor cells (CTCs) is a novel laboratory technique currently in use to determine response to therapy and to predict prognosis in breast, colorectal, and prostate cancer. We initiated a pilot study to analyze the role of CTCs as a biomarker of response and relapse in patients with EX-SCLC.

**Methods:** We collected blood samples from chemotherapy naïve patients with EX-SCLC prior to initiation of therapy, after completion of systemic therapy, and follow-up every 6–8 weeks and at relapse. The number of CTCs was determined using the cell search system in a central laboratory. The study was conducted in four different sites, and it was reviewed and approved by respective research review committees and IRBs.

**Results:** We enrolled 26 patients with EX-SCLC, 1 was excluded due to ineligibility, all were treated with platinum and etoposide. We observed partial response in 16 patients, stable disease in 3 patients, 1 patient with disease progression, and 6 patients were not assessed (5 deceased, 1 not available). The overall median number of CTCs in 24 patients measured at baseline and post-tx was 75 (range 0–3430) and 2 (range 0–526), respectively. A significant reduction in CTCs from baseline to post-treatment was identified for 15 subjects; the median reduction was 97.4% (range −100 to +100%, *p* < 0.001). Higher baseline CTCs and percentage change in post-treatment CTCs were associated with decreased survival.

**Conclusion:** We demonstrated that it is feasible to detect CTCs in EX-SCLC. If validated in other prospective studies, CTCs could be a useful biomarker in the management of EX-SCLC by predicting patients’ clinical responses to therapy.

## Introduction

Lung cancer is the leading cause of cancer death among men and women in the United States (1). Small-cell lung cancer (SCLC) is a variant of lung cancer marked by early metastases, and it accounts for 13% of all lung cancers diagnosed in the United States. At the time of presentation, two-thirds of patients will have disseminated disease making systemic chemotherapy the cornerstone of treatment. Sixty to eighty percent of patients with SCLC achieve an objective response with combination chemotherapy but despite these high responses, it is an aggressive disease with a median survival of 9–11 months for patients with extensive stage disease (EX-SCLC) (2). Clinical research efforts to improve the treatment for SCLC have been unsuccessful. Cisplatin or carboplatin and etoposide have been the treatment of choice for over 20 years (3). While we continue to pursue novel therapies for this disease, it is imperative that we concurrently pursue other strategies such as biomarker development to accurately monitor therapeutic responses, detect early progression, and predict clinical outcomes. Biomarkers are powerful tools to help us further understand the complex biology of cancer and to determine clinical responses in patients. Currently, there are no validated biomarkers to follow the disease activity in SCLC. Detection of circulating tumor cells (CTCs) is a novel laboratory technique currently in use to determine response to therapy and to predict prognosis in breast (4), colorectal (5), and prostate cancer (6). It is also present in lung cancer patients.

We hypothesize that CTCs will be a valuable and versatile biomarker for therapeutic response, determination of relapse, and survival in patients with SCLC. We conducted a study to determine if CTCs are a viable blood biomarker for survival, response, and relapse. We isolated and characterized CTCs using the CELLSEARCH^®^. This system is able to detect ≥2 CTCs in 36% of the 964 malignant samples compared to one (0.3%) of the 199 non-malignant samples and none of the 145 healthy controls (7).

The primary objective was to determine the number of CTCs in patients with EX-SCLC prior to initiation of chemotherapy, after therapy, and at time of relapse.

## Materials and Methods

### CTC detection

Patients were recruited from four participating sites: University of Kansas Medical Center, Kansas City VA, USA, University of Nebraska, and Omaha, VA, USA; the study was reviewed and approved by IRB of participating institutions. Eligible patients had a histological diagnosis of EX-SCLC and were planned to start chemotherapy. We collected blood samples for CTC detection before chemotherapy and within 4 weeks after treatment. The samples were collected from Monday to Wednesday in order to have time for processing. The CTC detection was performed centrally at University of Kansas Medical Center. We also obtained blood samples every 6–8 weeks during follow-up and at time of progression.

Sample preparation for isolation of epithelial cells from blood was done by collecting 7.5 mL of blood collected in special Veridex instrument tubes and mixed with 6.5 mL of buffer, centrifuged at 800 × *g* for 10 min, and then placed on the CellPrep system. After aspiration of the plasma and buffer layer by the instrument, ferrofluids were added, which were coated with epithelial cell-specific EpCAM antibodies that immunomagnetically isolated the epithelial cells from the patient sample (8). After the incubation period and subsequent magnetic separation, unbound cells and remaining plasma were aspirated and discarded. The staining reagents were then added in conjunction with a permeabilization buffer to fluorescently label the immunomagnetically isolated epithelial CTCs. After incubation on the system, the magnetic separation was repeated, and excess staining reagents were aspirated and discarded. In the final processing step, the cells were resuspended in the MagNest cell presentation device (Veridex LLC). This device consists of a chamber and two magnets that orient the immunomagnetically isolated and stained cells for analysis using the CellSpotter Analyzer.

### Sample analysis

The MagNest is placed on the CellSpotter Analyzer, a four-color semiautomated fluorescence microscope. Image frames covering the entire surface of the cartridge for each of the four fluorescent filter cubes are captured. The captured images containing objects that meet pre-determined criteria are automatically presented in a web-enhanced browser from which the final selection of cells is made by the operator. The criteria for an object to be defined as a CTC includes round to oval morphology, a visible nucleus (DAPI positive), positive staining for cytokeratin, and negative staining for CD45 (9, 10). Results of cell enumeration are always expressed as the number of cells per 7.5 mL of blood.

### Statistical considerations

This study was strictly exploratory and descriptive in nature. We intended to use the data to develop a larger, confirmatory study examining the association between CTC levels and survival, response, and relapse. The information in this study was to assist in deriving cutoff values for clinically meaningful differences in CTC levels from baseline to response and to relapse.

### Determination of sample size

Sample size calculations were based on a two-sided 95% confidence interval estimating the average drop in CTC levels from baseline to post-two cycles of treatment. For *n* = 25, a two-sided 95% confidence interval for the change would have an interval that extends no more than 0.916 from the observed difference in means with 80% coverage probability, assuming that the true standard deviation of differences is σ_d_ = 2 and that the confidence interval was based on the Student’s *t* statistic. To accommodate the possibility of dropout or samples that are not viable, an additional four (15%) subjects were enrolled, for a total sample size of *n* = 29. We anticipated the proportion of subjects who would respond to be 0.65.

## Results

We enrolled a total of 26 patients; one patient was not eligible because the patient had limited stage SCLC, for a total of 25 including 19 males (76%) and 6 females. The median age was 63 years old (range 50–79). All patients had extensive stage SCLC. The median pre-treatment CTC count was 75 (range 0–3430), and the median post-treatment CTC was 1 (range 0–526). Sixteen patients (61.5%) responded to therapy, 3 (11.5%) had stable disease, 1 (3.9%) experienced disease progression, and 6 (23.1%) patients did not have disease assessment.

Baseline characteristics of subjects were summarized using means and standard deviations for continuous variables; medians and ranges for continuous variables with skewness or outliers; and frequencies and percentages for categorical variables (Table 1). CTCs were summarized at baseline and at four post-treatment measurements using medians and ranges (Table 2). A description of the number of days lapsed between pre-treatment and each post-treatment measure is also provided. Percent change in CTCs from baseline was calculated as 100*(post – pre)/pre, and was summarized using medians and ranges. Non-parametric sign tests were used to test for significant decreases in CTCs from baseline levels (Table 2). Best response was summarized using frequencies and percentages. Overall survival (OS) was calculated as the number of subjects surviving at the end of the study period over the total number of study subjects. Kaplan–Meier product-limit estimates of survival were computed, and duration of survival was calculated as the time between baseline (pre-treatment) and date of death. Recurrence-free survival was calculated as the time between baseline (pre-treatment) and date of disease recurrence (Table 3). To test for an association between CTC levels and prognosis, we used Cox proportional hazards regression. The model included the covariates: baseline CTCs, percent-change in CTCs from baseline to post-treatment, and age (Table 4).

**Table 1:** **Baseline characteristics**.

	KCVA (*n* = 12)	KUCC (*n* = 7)	OMVA (*n* = 5)	UNMC (*n* = 2)	All Subjects (*n* = 26)
Age (mean, SD in years)^a^	62.6 (8.3)	60.8 (6.9)	68 (5.7)	70 (6.7)	63.8 (7.6)
Male Gender (*n*, %)^b^	11 (91.7)	2 (33.3)	5 (100)	1 (50)	19 (76)
CTCs (median, range)^c^	56 (0, 2835)	28 (1, 313)	257 (0, 1924)	1786.5 (143, 3430)	75 (0, 3430)

*^a^Age missing for one KUCC subject*.

*^b^Gender missing for one KUCC subject*.

*^c^Baseline CTCs missing for two subjects (KCVA and KUCC)*.

**Table 2:** **Circulating tumor cells detection**.

	*N*	Days since baseline^a^	CTCs^a^	% Change from baseline^a^	*p*^b^
Baseline	24	0	75 (0, 3430)	–	–
Post-Tx (1)	16	133 (69, 183)	1 (0, 526)	−98.7 (−100, 100)	<0.001
Post-Tx (2)	6	209.5 (177, 236)	7.5 (0, 1679)	−86.1 (−100, 2898)	0.7
Post-Tx (3)	2	266 (231, 301)	0.5 (0, 1)	−50 (−100, 0)	0.9
Post-Tx (4)	1	315 (–)	4 (–)	0 (–)	–

*^a^Median (minimum, maximum) reported*.

*^b^*p*-value is based on the non-parametric Sign test looking for a significant decrease from baseline levels*.

**Table 3:** **Response evaluation**.

	KCVA (*n* = 12)	KUCC (*n* = 7)	OMVA (*n* = 5)	UNMC (*n* = 2)	All subjects
Evaluable for response (*n* %)	9 (75)	5 (71.4)	5 (100)	1 (50)	20 (76.9)
Overall survival (*n* %)	0 (0)	3 (42.9)	1 (25)	0 (0)	4 (15.4)
Duration of survival (median, range in days)^a^	197 (14, 391)	252 (58, 385)	258.5 (185, 390)	201 (89, 313)	215 (14, 391)
**RESPONSE**					
Partial response (*n* %)	8 (66.7)	5 (71.4)	2 (40)	1 (50)	16 (61.5)
Stable disease (*n* %)	0 (0)	0 (0)	3 (60)	0 (0)	3 (11.5)
Progressive disease (*n* %)	1 (8.3)	0 (0)	0 (0)	0 (0)	1 (3.9)
Not evaluable (*n* %)	3 (25)	2 (28.6)	0 (0)	1 (50)	6 (23.1)
Recurrence-free survival (median, range in days)^b^	215 (66, 362)	273 (145, 497)	–	–	278 (147, 385)

*^a^Pre-treatment measurement to date of death, two censored subjects*.

*^b^Pre-treatment measurement to date of recurrence, three censored subjects*.

**Table 4:** **Maximum likelihood estimates (standard errors) and hazard ratios from cox proportional hazards regression model including the predictors baseline CTCs and % change from baseline to post-treatment**.

	MLE (SE)	χ^2^ (*p*)	HR
Baseline CTCs	0.025 (0.012)	4.4 (0.036)	1.025
% Change in CTCs	0.027 (0.016)	2.8 (0.09)	1.028

**Figure 1:**
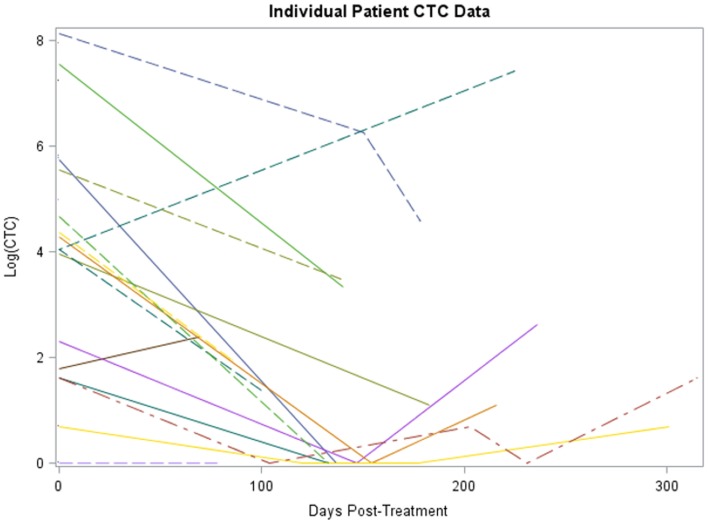
**Plot of individual patient-level CTC change data (log scale)**. The *x*-axis is number of days post-treatment at which the CTC measurement was collected for patient, *y*-axis is the percent change from baseline at that time for each patient, each patient has a line representing his or her change.

Twenty-four subjects had information on CTCs at baseline and 16 had at least one follow-up measurement post-treatment. The median number of days elapsed from baseline to post-treatment assessment was 135 (range 69–183 days). The median number of CTCs measured at baseline and post-treatment was 75 (range 0–3430) and 2 (range 0–526), respectively. A significant reduction in CTCs from baseline to post-treatment was identified for 16 subjects, the median reduction was 98.7% (range −100 to +100%, *p* < 0.001). A non-parametric sign test was used due to the small sample size and assumption violations of the parametric *t*-test. A plot of the individual patient data is provided in Figure 1.

**Figure 2:**
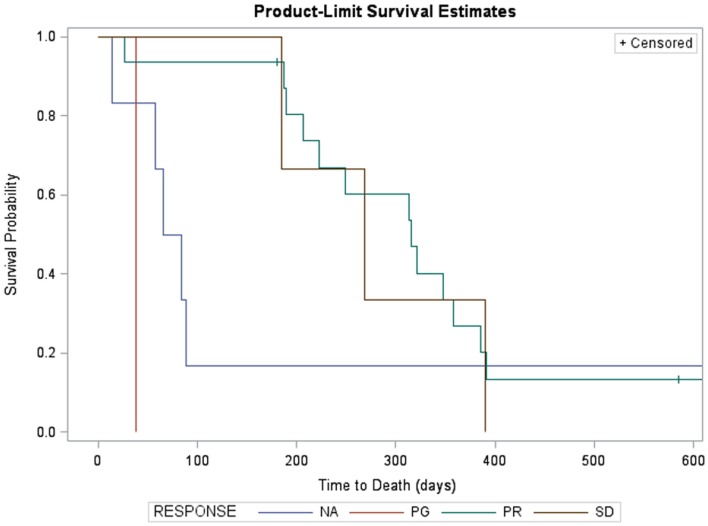
**Kaplan–Meier survival estimates of subjects who showed partial response (green, *n* = 12, two censored), progressive disease (red, *n* = 1), and those not evaluable for response due to early death (blue, *n* = 5)**. Three subjects with stable disease are not shown due to missing survival data. Significant differences in survival were found (log-rank χ^2^ = 25.3, *p* < 0.0001). Partial responders survived, on average, 321 days (95% CI: 189, 385). Subject with progressive disease survived 38 days. Subjects not evaluated for response had median survival of 65 days (95% CI: 14, 89).

Kaplan–Meier survival estimates of subjects who showed partial response, stable disease, progressive disease, and those not evaluable for response due to early death are displayed in Figure 2. Significant differences in survival were found (log-rank χ^2^ = 8.9, *p* = 0.03). Partial responders survived, on average, 315 days (95% CI: 207, 385). Subjects with stable disease had median survival of 268 days (95% CI: 185, 390). Subject with progressive disease survived 38 days. Subjects not evaluated for response had median survival of 74.5 days (95% CI: 14, 84).

**Figure 3:**
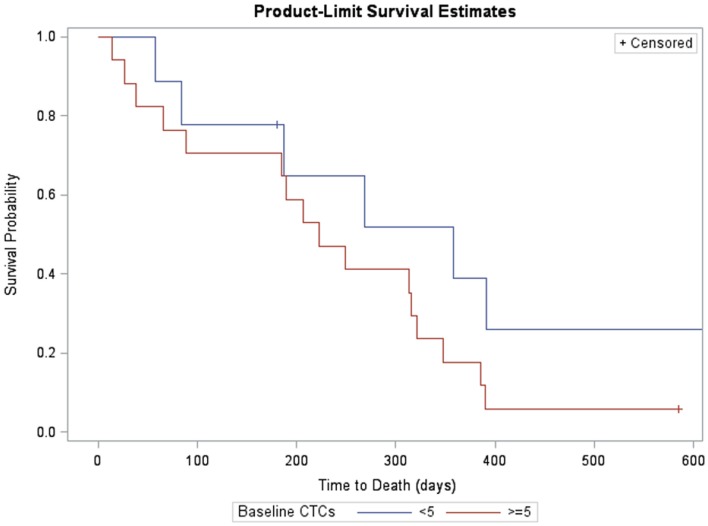
**Kaplan–Meier survival estimates of subjects with measurable CTCs at baseline (>5, red, *n* = 17, one censored) and without (<5. blue, *n* = 9, three censored)**. Subjects with >5 CTCs at baseline survived, on average, 223 days (95% CI: 65, 321). Subjects with <5 CTCs at baseline had median survival of 358 days (95% CI: 58, 358). Differences in survival were significant at the 10% level (log-rank χ^2^ = 1.99, *p* = 0.1).

Likewise, survival for subjects with fewer than five CTCs at baseline was compared with those with at least five CTCs (Figure 3). Subjects with ≥5 CTCs at baseline survived, on average, 223 days (95% CI: 65, 321). Subjects with <5 CTCs at baseline had median survival of 358 days (95% CI: 58, 358). Differences in survival were significant at the 10% level (log-rank χ^2^ = 1.99, *p* = 0.1).

Baseline CTCs (χ^2^ = 4.4, *p* = 0.036) and % change in CTCs from baseline to post-treatment (χ^2^ = 2.8, *p* = 0.09) was not statistically significant associated with survival but trended toward significance. Age was also not associated with survival (*p* > 0.1). On average, hazard ratios indicate the risk of death increases by 2.5% for every unit increase in CTCs at baseline, and by 2.8% on average for every unit increase in % change, that is, an increase of 10 CTCs at baseline is associated with a 25% increase in the risk of death (Table 4).

**Figure 4:**
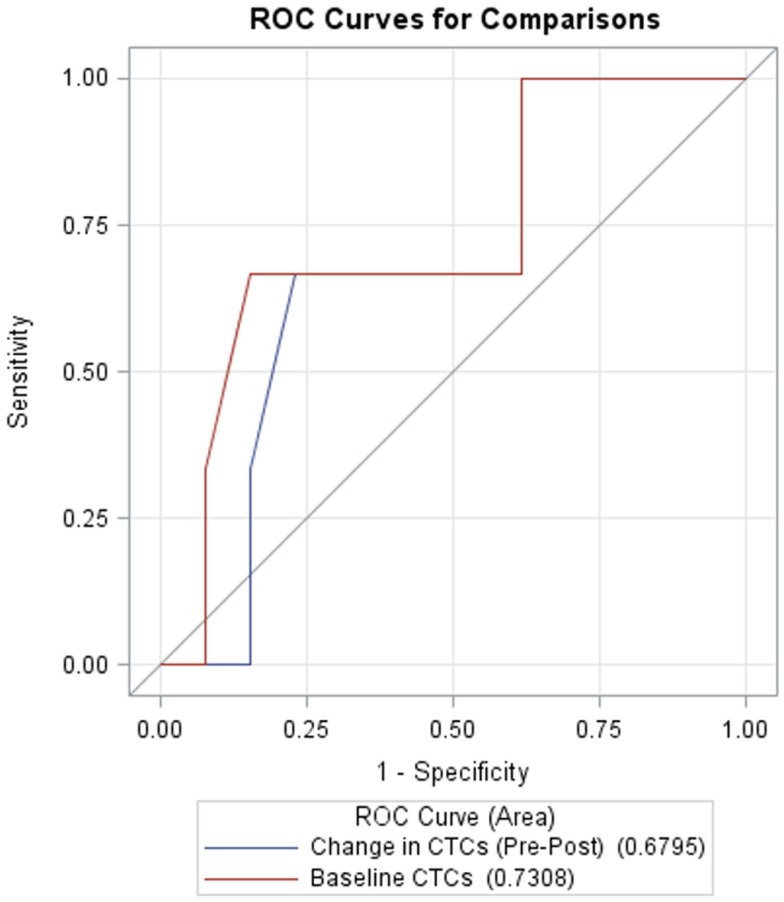
**Receiver operating characteristic (ROC) curves for CTCs at baseline (red, AUC = 0.7308, 95% CI: 0.36, 1.0) and change in CTCs from pre- to post-treatment (blue, AUC = 0.6795, 95% CI: 0.33, 1.0)**.

Receiver operating characteristic (ROC) curves for classifying survival as a function of baseline CTCs and pre- to post-treatment changes in CTCs are shown in Figure 4. AUC for baseline CTCs and change in CTCs are 0.731 (95% CI: 0.36, 1.0) and 0.68 (95% CI: 0.33, 1.0), respectively.

## Discussion

The presence of CTCs in the blood has been studied as a prognostic biomarker in several types of cancer. Cristofanilli et al. showed that CTCs are predictive of progression-free survival (PFS) and OS in 177 patients with metastatic breast cancer. Patients with levels of CTC ≥5 cells compared with patients with <5 CTCs had shorter median PFS (2.7 vs. 7 months, *p* < 0.001) and shorter OS (10.1 vs. 18 months *p* < 0.001). Patients with high versus low numbers of CTCs collected after the initiation of therapy had a PFS of 2.1 vs. 7 months (*p* ≤ 0.001) and OS of 8.1 vs. >18 months (*p* < 0.001) in their respective groups (4). CTCs have also been explored as predictors of treatment efficacy. Eighty-three untreated patients with metastatic breast cancer had their blood analyzed for detection of CTCs before treatment and then monthly for 6 months. Patients with ≥5 CTCs at baseline and at first follow-up (4 weeks) had a worse prognosis than patients with <5 CTCs. At baseline, the median PFS 4.9 vs. 9.5 months (*p* = 0.0014) and the median survival(S) was 14.2 vs. >18 months (*p* = 0.0048), respectively. At first follow-up, the median PFS was 2.1 vs. 8.9 months (*p* = 0.007) and median OS of 11.1 vs. >18 months (*p* = 0.0029), respectively (11).

Similarly, studies in prostate cancer have looked at CTCs as a prognostic marker in metastatic castration-resistant prostate cancer (CRPC) (6). At baseline, ≥5 CTCs corresponded to worsened median OS (21.7 vs. 11.5 months, *p* < 0.0001) in 231 patients. Patients with ≥5 CTCs 2–5 weeks after the initiation of treatment had a median OS 9.5 months, compared to a median OS of 20.7 months in patients with <5 CTCs. CTC counts predicted OS better than PSA decrement algorithms at all time points (*p* = 0.0218) (6).

The prognostic significance of utilizing CTCs was also demonstrated in metastatic colorectal patients. Cohen et al. studied presence of CTCs in 430 patients with mCRC at baseline and after starting first, second, or third line therapy. Patients were stratified into unfavorable and favorable prognostic groups based on CTC levels of ≥3 or <3 CTCs/7.5 mL, respectively. The unfavorable group had shorter median PFS (4.5 vs. 7.9 months; *p* = 0.0002) and OS (9.4 vs. 18.5 months; *p* < 0.0001) compared with favorable group (5). Based on these studies, the cell search system has been approved by FDA to predict PFS and OS in patients with metastatic breast cancer and monitoring of metastatic colorectal cancer and CRCP.

Similar to the results above, several reports in the literature along with our study showed that CTCs decreased in response to chemotherapy in SCLC supporting the use of CTCs as a biomarker for response. Naito et al. studied the presence of CTCs in patients with SCLC. There were 35 patients with detectable two or more baseline CTCs. Patients with ≥8 CTC levels at baseline had worse prognosis compared to patients with <8 CTC at baseline (*p* = 0.0014). Also, post-treatment CTCs ≥8 also had worse outcomes compared to <8. The study demonstrated that higher CTC count is associated with worse prognosis and it can be a valuable prognostic marker (12). A similar study by Hou et al. showed that CTCs were detectable in 77 of 97 patients. The study showed that pre-treatment CTC counts decrease after one cycle of chemotherapy, which is an independent prognostic factor. The OS was longer in patients with CTCs <50 compared to patients with CTC ≥50, 11.5 vs. 5.4 months, respectively (*p* < 0.0001) (13).

Our study only showed a trend between higher CTCs and percentage of change in post-treatment CTCs with worse outcome. This is probably because higher levels of CTCs at baseline would probably indicate a higher disease burden, and the degree of change of CTCs from baseline would probably indicate the efficacy of therapy. Our study is limited by the small sample size. A larger study is needed to confirm the link between the change of CTC and outcome.

Circulating tumor cells could potentially serve as a surrogate to the primary tumor. The future of utilizing CTCs would be greatly enhanced with genomic analysis of CTCs. The more we learn about CTCs, the more likely we will be able to use them in making bedside treatment decisions.

## Conclusion

The identification of CTCs in blood samples of patients with EX-SCLC is feasible in this multi-institutional pilot study. A larger trial is necessary to study its prognostic significance. It is possible that in the future, CTCs will be incorporated in the management of SCLC and be used to monitor early recurrence. This could allow us to make treatment decisions before there is evidence of radiographic disease in order to help suppress clinical progression which could lead to extended survival.

## Conflict of Interest Statement

The study was conceptualized and conducted by the investigator without the influence of the funding source. Funding provided in part by Veridex. Veridex provided the kits needed for the assay of the CTCs. The institution and the investigator did not receive payment or have financial interest in the company. There are no copyrights or royalties relevant to this work.

## Supplementary Material

The Supplementary Material for this article can be found online at http://www.frontiersin.org/Journal/10.3389/fonc.2014.00271/abstract
