# Supplementary material for: A Multicenter Pilot Study Examining the Role of Circulating Tumor Cells as a Blood-Based Tumor Marker in Patients with Extensive Small-Cell Lung Cancer
Source: Front Oncol. 2014 Oct 14;4:271. doi: 10.3389/fonc.2014.00271 (PMC4196518; doi:10.3389/fonc.2014.00271)
Supplement: Supplementary file 1 [file Table1.DOCX]

| **Table S1 supplemental data of CTC pre and post therapy** | | | | | | | | |  |  |  |
| --- | --- | --- | --- | --- | --- | --- | --- | --- | --- | --- | --- |
| **Site** | **PT ID** | **Pre-treatment CTC** | **Date** | **Post-Treatment CTC** | **Response** | **Date of Death** | **Survival Time (Days)** | **Notes** | | |  |
| OMVA | 5536 | 0 | 10/16/2012 | NA | SD | NA | NA |  | | |  |
| KCVA | 3836 | 0 | 4/11/2012 | NA | PR | 5/7/2013 | 386 |  |  |  |  |
| KCVA | 5391 | 0 | 9/26/2012 | 0 | PR | 4/9/2013 | 193 |  |  |  |  |
| KUCC | 2522 | 1 | 9/16/2010 | NA | NA | 11/13/2010 | 57 |  |  |  |  |
| KUCC | 3487 | 1 | 1/25/2012 | 0 | PR | 5/14/2014 | 829 | Last contact 5/14/14 | | |  |
| KUCC | 2582 | 4 | 11/11/2010 | 0 | PR | 6/10/2011 | 209 |  | | |  |
| KCVA | 2606 | 4 | 12/15/2010 | 0 | PR | 12/8/2011 | 353 |  |  |  |  |
| KCVA | 2603 | 5 | 12/9/2010 | 10 | PR | 7/4/2011 | 205 |  |  |  |  |
| KCVA | 2628 | 9 | 12/22/2010 | 0 | PR | 11/8/2011 | 316 |  |  |  |  |
| KUCC | 2742 | 52 | 3/7/2011 | 2 | PR | 4/13/2012 | 396 |  |  |  |  |
| KCVA | 3907 | 56 | 5/2/2012 | 3 | PR | 12/11/2012 | 219 |  |  |  |  |
| KCVA | 3197 | 71 | 9/8/2011 | 0 | PR | 11/30/2011 | 82 |  |  |  |  |
| KUCC | 5466 | 79 | 7/9/2012 | 6 | PR | 6/15/2013 | 336 |  |  |  |  |
| OMVA | 5305 | 106 | 9/13/2012 | NA | PR | NA | NA |  |  |  |  |
| KCVA | 2776 | 143 | 3/22/2011 | NA | NA | 5/26/2011 | 64 |  |  |  |  |
| UNMC | 3477 | 143 | 1/18/2012 | NA | NA | 4/16/2012 | 88 |  |  |  |  |
| OMVA | 4092 | 257 | 9/14/2011 | 32 | PR | NA | NA |  |  |  |  |
| KUCC | 2508 | 313 | 9/3/2010 | 0 | PR | 3/11/2011 | 188 |  |  |  |  |
| OMVA | 4121 | 319 | 9/19/2011 | 19 | SD | 3/22/2012 | 183 |  |  |  |  |
| KCVA | 2869 | 331 | 4/20/2011 | NA | PR | 5/28/2011 | 38 |  |  |  |  |
| KCVA | 3937 | 1373 | 5/9/2012 | NA | NA | 5/23/2012 | 14 | Instrument failure | | |  |
| OMVA | 3417 | 1924 | 12/29/2011 | 27 | SD | NA | NA |  | | |  |
| KCVA | 4215 | 2835 | 11/3/2011 | NA | PR | NA | NA | Excluded-did not meet eligibility | | | |
| UNMC | 3703 | 3430 | 3/12/2012 | 526 | PR | NA | NA |  | | |  |
| KCVA | 3984 | NA | 5/17/2012 | NA | NA | NA | NA | Blood clotted | | |  |
| KCVA | 4288 | NA | 5/31/2012 | NA | NA | NA | NA | Instrument failure | | |  |
|  |  |  |  |  |  |  |  | NA – Not available | | |  |
